# Population‐Level Laterality of Ambush Posture in the Chinese Green Tree Pit Viper ( *Trimeresurus stejnegeri* ) in Northern Taiwan

**DOI:** 10.1002/ece3.73627

**Published:** 2026-05-05

**Authors:** Hung‐Chia Chang, Si‐Min Lin

**Affiliations:** ^1^ School of Life Science, National Taiwan Normal University Taipei Taiwan

**Keywords:** ambush posture, behavioral lateralization, citizen science, pit viper, sit‐and‐wait predation, *Trimeresurus stejnegeri*

## Abstract

Behavioral lateralization, a consistent directional asymmetry in behavior, has been documented across many vertebrates, yet population‐level evidence in snakes remains limited and debated. We examined ambush‐posture laterality in the Chinese green tree pit viper ( *Trimeresurus stejnegeri* ), a nocturnal sit‐and‐wait predator, using a large dataset compiled from nocturnal field surveys (2014–2025) and verified citizen‐science records from iNaturalist (2013–2025). Ambush‐posture direction was classified from photographs using a standardized, anatomically referenced procedure based on head‐aligned reference lines, and ambiguous cases were excluded unless observer classifications were concordant. We tested for population‐level bias among sites using chi‐squared tests and binomial logistic regression, and evaluated effects of age class and perching height. Across 283 observations from three localities, snakes at Yangmingshan exhibited a significant rightward bias in ambush posture (36 of 55 individuals = 65.5%; *χ* ^2^ = 5.25, df = 1, *p* = 0.022), whereas no population‐level bias was detected at the other two localities. Neither age class nor perching height significantly affected laterality. These results provide the first evidence for site‐specific population‐level ambush‐posture laterality in a snake and suggest that local ecological or demographic contexts, potentially linked to prey community composition and availability, may modulate the expression of laterality. Future work across additional populations and taxa, together with performance‐based tests comparing capture success between left‐ versus right‐curved strikes, will be essential for evaluating the adaptive significance of this pattern.

## Graphical abstract

In the Chinese green tree pit viper (*Trimeresurus stejnegeri*), we tested whether ambush posture shows population‐level laterality using 283 photographic records from nocturnal field surveys and verified iNaturalist observations across three regions in northern Taiwan. One focal population showed a significant rightward bias, while the other sites did not. Our results suggest that ambush‐posture laterality in snakes can be locally expressed and shaped by population‐specific contexts.

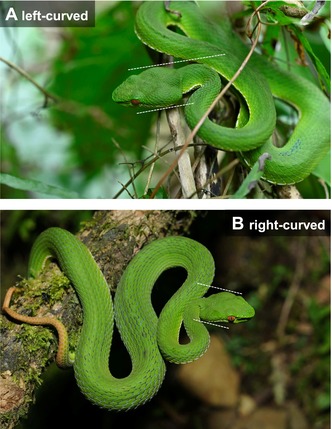

## 1 Introduction

Lateralization refers to consistent behavioral or functional biases displayed by organisms and is most commonly attributed to cerebral lateralization, which involves asymmetrical processing of cognitive functions between the two brain hemispheres. A well‐known example is handedness in humans and great apes (Hopkins 2006; Cashmore et al. 2008). Cerebral lateralization is thought to enhance cognitive efficiency and multitasking ability and has been linked to fitness‐related traits such as social interactions, foraging efficiency, and predator avoidance (Berlinghieri et al. 2021). Behavioral lateralization has been documented across a wide range of vertebrate taxa, including fishes, amphibians, reptiles, birds, and mammals (Lippolis et al. 2002; Brown et al. 2004; Amano et al. 2021; Karenina and Giljov 2022; Honan and Murray 2023).

Current studies on laterality in snakes have primarily focused on how morphological asymmetries may influence behavioral lateralization (Shine et al. 2000; Chang et al. 2021). For instance, Shine et al. (2000) reported that male garter snakes ( *Thamnophis sirtalis* ) possess asymmetrical hemipenes and gonads. At higher temperatures, snakes tend to use the larger hemipenis, likely because the larger one and its associated gonad contain more sperm. However, at lower temperatures, the larger hemipenis is more difficult to adjust into the appropriate mating position, making it less frequently used. Other studies focused on anti‐predatory behaviors, such as the directional preference in coiling (clockwise vs. counterclockwise), leading to conflicting conclusions (Roth 2003; Heatwole et al. 2007). Roth (2003) first suggested lateralized coiling behavior in 3 of 30 *Agkistrodon piscivorus* individuals within the population, showing individual and also population level of laterality. In contrast, Heatwole et al. (2007) reported that coiling‐direction laterality was not supported in their periodic records of copperheads ( *Agkistrodon contortrix* ) and cottonmouths ( *A. piscivorus* ). Neither species showed a significant population‐level preference, and only 1 of 22 individuals exhibited a significant bias. They noted that this proportion falls within chance expectations when hypothesis tests are evaluated at a 5% rejection threshold. Since the earlier report of individual‐ and population‐level laterality was not replicated by the later and more extensive study, the existence and functional significance of behavioral laterality in snakes remain debated.

Most work on snake laterality has focused on coiling, so we asked whether a population‐level signal could be detected in ambush posture during feeding. In sit‐and‐wait predators, ambush posture might directly affect strike direction and prey‐capture performance, making it a functionally relevant context in which to test for lateralized behavior. Here, we tested whether ambush‐posture direction in the Chinese green tree pit viper ( *Trimeresurus stejnegeri* ) shows detectable population‐level laterality in northern Taiwan, and whether any such pattern varies among sites and age classes. We further examined whether ambush laterality varies among sites and microhabitats, because habitat differences may be associated with differences in prey composition, and variation in food sources has been linked to lateralized behavior in other taxa (Friedlaender et al. 2017; Karenina and Giljov 2022). We also tested whether laterality differs between age classes, as age‐related variation in lateralization has been reported previously (Uziel et al. 1998; Morita et al. 2023).

## 2 Methods

### 2.1 Study Species

Chinese green tree pit viper ( *Trimeresurus stejnegeri* ) is a nocturnal, venomous ambush predator that employs a sit‐and‐wait foraging strategy, making it well suited for studies of behavioral laterality during ambush posture. The species is widely distributed across Taiwan, eastern and southern China, northern Vietnam, and northeastern Laos, and is one of the most abundant snake species in Taiwan, allowing robust population‐level analyses. It is an arboreal, euryphagous dietary generalist that feeds primarily on amphibians, but also consumes reptiles, birds, mammals, and occasionally insects (Tsai and Tu 2000; Creer et al. 2002; Yang and Mori 2021; Xu et al. 2025).

### 2.2 Data Collection and Filtering

Ambush‐posture observations were compiled from two sources: nocturnal field surveys of HCC conducted between 2014 and 2025 (Table A1), and all available citizen‐science records from iNaturalist collected between 2013 and 2025 (Table A2; accessed 27 November 2025).

Field surveys were conducted as nocturnal visual encounter surveys (4–6 h per session), and all encountered individuals of *T. stejnegeri* were recorded. Observations were collected from three regions in northern Taiwan: (1) Tianmu Hiking Trail, Shilin District, Taipei City (centered at 25.131772, 121.535549; hereafter “Tianmu” or abbreviated as TM); (2) Bailaka Road from Beitou District, Taipei City to Sanzhi District, New Taipei City, including adjacent region within Yangmingshan National Park (centered at 25.184430, 121.516428; hereafter “Yangmingshan” or abbreviated as YM); and (3) Northern Cross‐Island Highway from Baling, Taoyuan City to Mingchi, Yilan County (centered at 24.647897, 121.429505; hereafter “Highway” or abbreviated as HW). Survey effort was summarized as total transect distance and number of survey sessions at each site: Tianmu (1.6 km, *n* = 6), Yangmingshan (9.3 km, *n* = 17), and Highway (16.6 km, *n* = 14). Photographs were taken in the field for subsequent classification of ambush‐posture direction, which yielded 175 observations. Among these, 54 were excluded because the snakes were moving or resting (*n* = 45), or photographed at an angle unsuitable or ambiguous for determining ambush laterality (*n* = 9). The remaining 121 field‐survey records were retained for laterality scoring (Table A1).

For iNaturalist observations, site was assigned based on the observation locality and geographic coordinates provided with each record, and records were grouped into Tianmu, Yangmingshan, or Highway. We found a total of 362 records from these three sites, of which 187 were excluded because they did not represent undisturbed ambush posture. Specifically, individuals were removed if they were feeding, mating, dead, potentially disturbed by observers (total *n* = 150), or ambiguous for determining ambush laterality (see below, *n* = 37). To minimize pseudoreplication, same‐day observations from the same location by different observers were screened; repeated records of the same individual were removed. Snakes were identified as the same individual based on their unique scars, color pattern, body injuries, or identical ambush postures at the same location. When duplicates occurred (*n* = 13), the earliest citizen‐science photograph was retained. After filtering, laterality frequencies did not differ between field surveys (*n* = 121, Table A1) and citizen‐science records (*n* = 162, Table A2) (*χ* ^2^ = 0.10, df = 1, *p* = 0.756), allowing the datasets to be pooled for subsequent analyses.

All data were obtained through non‐invasive observations of free‐ranging snakes. Since no experimental procedures were conducted, no animal ethics approval was required in this study.

### 2.3 Determination of Ambush Laterality

**FIGURE 1:**
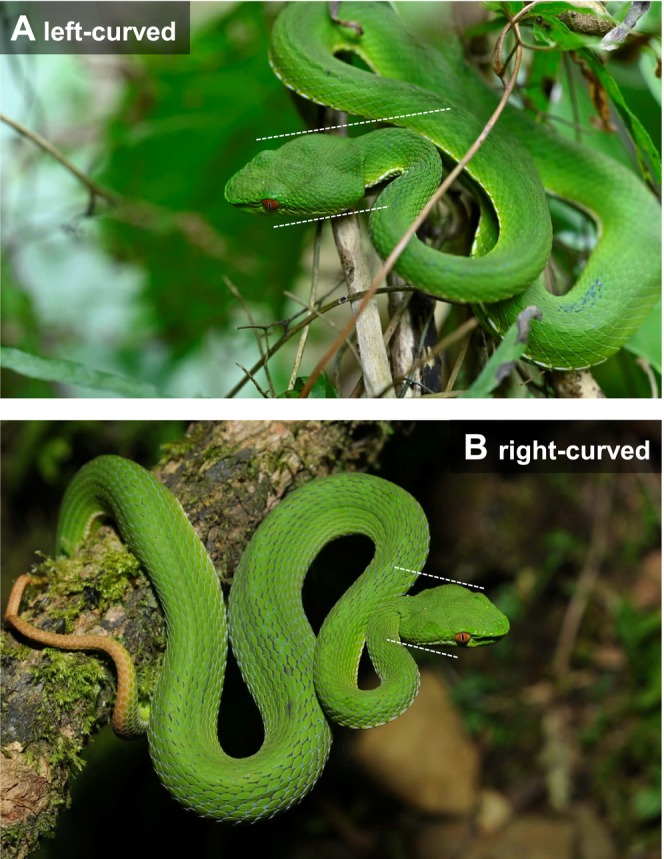
Ambush posture of the Chinese green tree pit viper ( *Trimeresurus stejnegeri* ). (A) Left‐curved ambush posture, and (B) right‐curved ambush posture. Reference lines are used to classify posture directions. During the initial straightening phase of a strike, a right‐curved posture would be expected to involve greater activation of the right‐side neck muscles (with relative relaxation on the left), whereas a left‐curved posture would show the opposite pattern. Photographed by Yu‐Jhen Liang.

Ambush posture was identified when a snake was stationary with the forebody arranged in a concertina‐like shape, following Shine and Sun (2003). Ambush posture direction was classified from photographs using a standardized procedure. We identified the most laterally expanded points of the temporal venom glands (one on each side of the head) and drew two reference lines parallel to the longitudinal axis of the head passing through these points. Posture direction was then determined by the side of the body that first intersected either reference line: intersection with the snake's right body side indicated a right‐curved posture, whereas intersection with the left body side indicated a left‐curved posture (from the snake's perspective; Figure 1). To minimize observer bias, all photographs were independently classified by HCC and SML in a blind manner. Records were considered ambiguous when the photographic angle prevented the reference‐line method from being applied reliably. Such ambiguous records (9 from field surveys and 37 from citizen‐science observations) were excluded from the final analyses.

### 2.4 Age Class, Microhabitat, and Seasonal Assignment

Because many individuals in this study were identified solely from photographs, age class was inferred from relative morphological proportions rather than absolute size. Previous studies have shown ontogenetic allometric changes in the eyes and head of *Trimeresurus* snakes, such that adults have proportionally smaller eyes and smaller heads than juveniles (Tsai and Tu 1998; Herrel et al. 2011; Huang et al. 2022). To quantify these relative differences, we measured a set of individuals of known age class and calculated the eye diameter/head length ratio, with head length measured from the tip of the snout to the posterior edge of the venom gland. Juveniles generally showed eye diameter/head length ratios greater than 0.139 (mean ± SE = 0.173 ± 0.029, range = 0.139–0.241, *n* = 30), whereas adults had ratios below 0.139 (mean ± SE = 0.117 ± 0.013, range = 0.083–0.135, *n* = 30). Based on this reference dataset, we used ImageJ to assign individuals with an eye diameter/head length ratio greater than 0.139 to the juvenile class as a photograph‐based operational proxy; individuals with lower ratios were classified as adults.

Perching height was categorized as “high” or “low” based on whether the snake was plausibly positioned to strike at ground‐dwelling prey. Low perching sites included individuals positioned on the ground, at the base of tree trunks, or on steep slopes, but only when the body was oriented toward the ground and the vertical distance to the ground was within a plausible strike range relative to body length (< 1/2 body length). High perching sites included individuals on vegetation, fences, or other elevated structures from which attacks on prey on the ground were unlikely. Records were excluded when the ground surface was not visible and perching height could not be assigned reliably (*n* = 12). Seasons were defined according to Taiwan's climatic divisions: spring (March–May), summer (June–August), autumn (September–November), and winter (December–February).

### 2.5 Statistics

Differences in laterality between locations, perching height, and age classes were tested using chi‐squared tests of homogeneity. Within each category, chi‐squared goodness‐of‐fit tests were used to evaluate whether left–right frequencies deviated from a 1:1 expectation. Because the dataset contained sufficiently large sample sizes, chi‐squared tests were appropriate and provided reliable approximations of expected distributions. For descriptive purposes, we also calculated a proportional laterality index modified from Roth (2003): Laterality index = *R*/(*R* + *L*), where *R* and *L* are the numbers of right‐ and left‐biased individuals, respectively. In addition, we tested whether each age class exhibited a preference for perching height (“low” vs. “high”) using chi‐squared goodness‐of‐fit tests. Differences in perching height distribution between adults and juveniles were evaluated using a chi‐squared test of independence.

To identify predictors of ambush laterality, we initially fitted a full generalized linear model (GLM) with a binomial error structure and logit link. Laterality (0 = left, 1 = right) was used as the response variable. The full model included all main effects (age class, perching height, site, season, data source, and year) and biologically plausible interactions (height × site and height × age), allowing us to test whether the effect of perching height on laterality varied among sites or age classes. We then performed stepwise model simplification based on likelihood ratio tests and evaluated model fit using Akaike's information criterion (AIC). During this process, the data source variable remained in the model (AIC = 380.22). To directly address our biological hypotheses, we also fitted a reduced model including only the three predictors of primary interest (site, perching height, and age class). This model had the lowest AIC (378.75) and provided the most parsimonious and interpretable explanation of variation in laterality. Therefore, this reduced model was selected as the final model.

For significant predictors, we conducted post hoc pairwise comparisons using chi‐squared tests with Holm–Bonferroni correction for multiple testing. Because site was identified as a significant predictor, we further performed site‐specific analyses to evaluate whether age class, data source, year, season, and perching height were associated with laterality within the site showing a significant effect. These analyses were conducted using logistic regression (GLM with binomial error structure). Predicted probabilities of right‐side lateralization were calculated for each individual based on the fitted models. Records with missing values in any variables were excluded from all analyses. All statistical analyses were conducted in R 4.4.1, with significance assessed at *α* = 0.05.

## 3 Results

Among all observations (Table 1), 149 of 283 snakes (52.7%) showed right‐curved posture and 134 left‐curved (47.3%) (*χ* ^2^ = 0.80, df = 1, *p* = 0.373). Adults (right‐curved 52.5%; *χ* ^2^ = 0.60, df = 1, *p* = 0.440, *n* = 242) and juveniles (right‐curved 53.7%; *χ* ^2^ = 0.22, df = 1, *p* = 0.639, *n* = 41) were both unbiased, and no difference was detected between age classes (*χ* ^2^ = 0.02, df = 1, *p* = 0.889). Likewise, no lateral bias was found in either low (53.6%; *χ* ^2^ = 1.15, df = 1, *p* = 0.283, *n* = 222) or high perching sites (49.0%; *χ* ^2^ = 0.02, df = 1, *p* = 0.886, *n* = 49), and there was no difference between these two groups (*χ* ^2^ = 0.11, df = 1, *p* = 0.743).

**TABLE 1:** Retained sample sizes, data source, with counts of left‐curved and right‐curved ambush postures (*n* = 283).

Site	Data source	Effective *n*	Left‐curved	Right‐curved	Left‐curved	Right‐curved
Tianmu Hiking Trail (TM)	Field survey	16	11	5	19	18
	iNaturalist	21	8	13		
Yangmingshan National Park (YM)	Field survey	17	7	10	19	36
	iNaturalist	38	12	26		
Northern Cross‐Island Highway (HW)	Field survey	88	38	50	96	95
	iNaturalist	103	58	45		
Total		283			134	149

**FIGURE 2:**
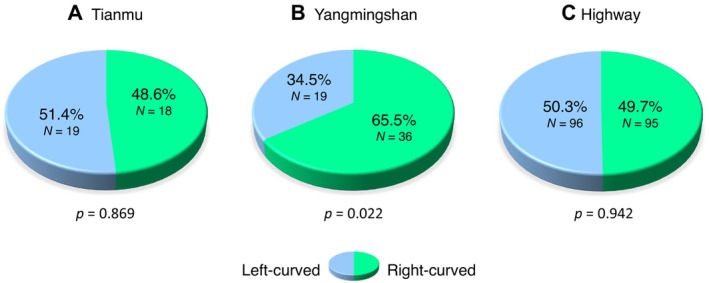
Laterality of ambush posture in the Chinese green tree pit viper ( *Trimeresurus stejnegeri* ) across different locations. The Yangmingshan population reveals significant deviations from a 1:1 left–right expectation (*p* = 0.022, chi‐squared goodness‐of‐fit test).

At the locality level, no bias was detected at Tianmu (right‐curved 48.6%; *χ* ^2^ = 0.03, df = 1, *p* = 0.869) and Highway (right‐curved 49.7%; *χ* ^2^ = 0.01, df = 1, *p* = 0.942). In contrast, snakes at Yangmingshan showed a significant bias in ambush posture, with 36 of 55 individuals (65.5%) being right‐biased (*χ* ^2^ = 5.25, df = 1, *p* = 0.022) (Figure 2). However, laterality did not differ significantly among the three localities when tested simultaneously (*χ* ^2^ = 4.50, df = 2, *p* = 0.105). This pattern reflects limited among‐site heterogeneity, as frequencies at Tianmu and Highway were close to a 1:1 ratio.

In the three‐factor GLM (i.e., age, perching height, site), only the Yangmingshan site significantly predicted lateralization (Table 2, *p* = 0.029, *n* = 271), with individuals more likely to be right‐curved. Age class and perching height were not significant predictors (Table 2, *p* > 0.05, *n* = 271). However, post hoc pairwise comparisons revealed no significant differences between Yangmingshan and Highway (*χ* ^2^ = 4.24, df = 1, *p* = 0.079) or between Yangmingshan and Tianmu (*χ* ^2^ = 2.58, df = 1, *p* = 0.108). A site‐specific GLM for Yangmingshan revealed no significant predictors of right‐curved lateralization (Table A3; *p* > 0.05, *n* = 51).

**TABLE 2:** Generalized linear model (GLM) on ambush laterality in *Trimeresurus stejnegeri* (*n* = 271).

Variables	Estimate	SE	*z*	*p*	95% CI
Intercept	−0.0137	0.156	−0.088	0.930	−0.319, 0.291
Age (juvenile vs. adult)	0.2608	0.365	0.715	0.474	−0.454, 0.976
Perching height (high vs. low)	−0.2223	0.356	−0.624	0.532	−0.920, 0.476
Site (TM vs. HW)	−0.2156	0.388	−0.555	0.579	−0.977, 0.545
Site (YM vs. HW)	0.7569	0.346	2.188	0.029*	0.079, 1.435

Abbreviations: HW, Northern Cross‐Island Highway; TM, Tianmu; YM, Yangmingshan.

* *p* < 0.05.

An additional ecological pattern was observed regarding perching height. Adult snakes exhibited a significant preference for low perching heights (87.0%; *χ* ^2^ = 125.65, df = 1, *p* < 0.001, *n* = 230), whereas juveniles showed no such preference, with 51.2% utilizing low perches (*χ* ^2^ = 0.02, df = 1, *p* = 0.876, *n* = 41). Consequently, the distribution of perching heights differed significantly between life stages (*χ* ^2^ = 29.54, df = 1, *p* < 0.001).

## 4 Discussion

In this study, Chinese green tree pit vipers exhibited a significant right‐curved bias in ambush posture at the Yangmingshan site, whereas no lateral bias was detected at the other two localities. Neither age class nor perching height was associated with laterality. To our knowledge, this is the first report of site‐specific population‐level ambush‐posture laterality in a snake, suggesting that such laterality, if present, may be expressed only under certain local conditions.

Predator lateralization can evolve in response to lateralized prey, particularly when prey asymmetries consistently bias the direction of escape or handling. Such prey‐driven asymmetry has been documented in snakes, for example in snail‐specialist *Pareas* spp., where prey morphology is linked to dentition asymmetry and directional biases in feeding behavior of the snakes (Hoso et al. 2007; Hoso 2017; Chang et al. 2021). Because amphibians can exhibit lateralized escape responses (Dill 1977; Anderson et al. 2021; Keuroghlian‐Eaton et al. 2024), we propose as a first possibility that the Chinese green tree pit viper, a predominantly frog‐eating predator (Creer et al. 2002; Yang et al. 2024), may show a corresponding lateralization in ambush posture that influences strike direction and capture success. Yangmingshan is also regarded as a locality with relatively high amphibian diversity and abundant frog resources (Poo et al. 2003; You and Wang 2007; Chen 2023). We therefore consider it possible that greater availability of frog prey may contribute to the site‐specific pattern observed in this population.

Another speculative possibility is that hemispheric specialization could contribute to consistent side biases in strike‐related posture. During a strike initiated from an ambush posture, the snake rapidly straightens from a lateral bend, with stronger activation occurring primarily in the muscles on the concave (bent) side (Jayne 2020). For example, in the Chinese green tree pit viper, a right‐curved individual would be expected to contract the neck muscles on the right side (with relative relaxation on the left) during the initial straightening phase of a strike, whereas a left‐curved individual would show the opposite pattern. In vertebrates, the left hemisphere is often associated with directing attention toward specific categories of stimuli and with controlling feeding‐related responses (Robins and Rogers 2004, 2006; Rogers 2021). Although we did not measure neural or muscular activity directly, such hemispheric specialization could be consistent with the predominance of right‐curved ambush postures observed in our study if it makes one side of the body more likely to be activated during strikes (Figure 1). Similar lateralized feeding behaviors have been reported in other reptiles, including the common wall lizard ( *Podarcis muralis* ) (Bonati et al. 2008; Csermely et al. 2010) and the ornate dragon lizard ( *Ctenophorus ornatus* ) (Robins et al. 2005). Differences in the frequency of lateralized individuals among populations have also been documented (e.g., in humans, where handedness varies among localities; Singh and Bryden 1994; Raymond and Pontier 2004), which may help explain why ambush laterality was evident at Yangmingshan but not at the other study sites.

Snakes exhibit internal anatomical asymmetry, most notably in the lungs, with the right lung being larger than the left (van Soldt et al. 2015). This visceral asymmetry could provide a mechanistic basis for a rightward bias in posture. When a snake coils or adopts an S‐shaped pre‐strike posture, the larger right lung may be positioned to reduce compressive forces. In addition, the non‐circular cross‐section of the body may create side‐specific differences in compression and tension. Roth (2003) reported that all *Agkistrodon piscivorus* individuals expressing laterality in his study coiled clockwise, with the larger right lung positioned on the outer side of the coil. Although one *A. contortrix* individual showed the opposite coiling direction (counterclockwise), that species did not exhibit population‐level lateralization (Heatwole et al. 2007). Thus, the relationship between visceral asymmetry and behavioral biases remains unresolved.

In addition to laterality, we observed an ontogenetic difference in microhabitat use. Adult snakes showed a clear preference for low perching sites, whereas juveniles did not exhibit a significant preference. A similar pattern has been reported by Yang et al. (2024), in which adults primarily used lentic water bodies as ambush sites, whereas juveniles used multiple habitat types with comparable frequency. This pattern suggests that microhabitat use may shift with age, potentially reflecting differences in experience or foraging strategy. However, because perching height was not associated with ambush laterality in our analyses, this ontogenetic shift is unlikely to explain the observed site‐specific lateralization.

Our results do not indicate a general, species‐wide pattern of ambush laterality in *T. stejnegeri* . Instead, a significant right‐curved bias was detected only in the Yangmingshan population, whereas the other two localities remained close to a 1:1 ratio. This suggests that ambush posture laterality, if present, may be locally expressed rather than fixed across populations, and that any anatomical predisposition may become detectable only under certain ecological or demographic conditions. Determining whether this site‐specific pattern reflects differences in prey communities, habitat structure, sampling effects, or stochastic variation will require replication across additional localities and populations. Broader comparative work will help clarify when and why visceral asymmetry translates into consistent behavioral lateralization. Finally, evaluating the adaptive significance of lateralized ambush posture will require direct tests of foraging performance, particularly comparisons of capture success (or prey escape probability) between strikes initiated from left‐ versus right‐curved postures.

## Author Contributions

**Hung‐Chia Chang:** conceptualization (equal), data curation (lead), formal analysis (lead), investigation (lead), methodology (equal), validation (equal), visualization (supporting), writing – original draft (equal), writing – review and editing (supporting). **Si‐Min Lin:** conceptualization (equal), funding acquisition (lead), methodology (equal), resources (lead), supervision (lead), validation (equal), visualization (lead), writing – original draft (equal), writing – review and editing (lead).

## Funding

This work was supported by the National Science and Technology Council (MOST 111‐2621‐B‐003‐001‐MY3, NSTC 112‐2621‐B‐003‐002‐MY3).

## Conflicts of Interest

The authors declare no conflicts of interest.

## Data Availability

All data are available in the main text and Appendix A.

## Appendix A

**TABLE A1:** Field observations for *Trimeresurus stejnegeri* laterization, with ambush‐posture direction classified as left‐curved (L) or right‐curved (R).

No.	Year/Month/Date	Laterality	No.	Year/Month/Date	Laterality
**Tianmu Hiking Trail (TM) (*n* = 16)**			**Yangmingshan National Park (YM) (*n* = 17)**		
1	2018/03/31	R	1	2018/11/12	L
2	2018/03/31	R	2	2020/06/13	L
3	2018/03/31	L	3	2021/07/17	R
4	2019/03/12	L	4	2021/10/10	R
5	2019/03/12	L	5	2022/05/13	R
6	2020/03/08	R	6	2022/05/13	L
7	2020/03/08	R	7	2022/06/19	R
8	2020/03/08	L	8	2025/04/12	R
9	2020/03/08	L	9	2025/04/12	R
10	2020/03/08	L	10	2025/04/23	L
11	2020/03/08	L	11	2025/05/31	R
12	2022/04/08	R	12	2025/11/16	R
13	2022/04/08	L	13	2025/11/16	R
14	2025/09/22	L	14	2025/11/16	L
15	2025/09/22	L	15	2025/11/16	R
16	2025/09/22	L	16	2025/11/16	L
			17	2025/11/16	L
**Northern Cross‐Island Highway (HW) (*n* = 88)**					
1	2014/09/27	R	45	2014/11/08	L
2	2014/09/27	R	46	2014/11/08	L
3	2014/09/27	R	47	2014/11/08	R
4	2014/09/27	R	48	2015/11/21	L
5	2014/09/27	R	49	2015/11/21	L
6	2014/09/27	R	50	2018/04/22	R
7	2014/09/27	L	51	2018/04/22	R
8	2014/09/27	R	52	2018/04/22	R
9	2014/09/27	L	53	2018/05/18	L
10	2014/09/27	R	54	2018/10/20	R
11	2014/09/27	R	55	2018/10/20	L
12	2014/09/27	L	56	2018/10/20	L
13	2014/09/27	L	57	2018/10/20	R
14	2014/09/27	R	58	2018/10/20	R
15	2014/09/27	L	59	2018/10/20	R
16	2014/09/27	L	60	2018/10/20	R
17	2014/09/27	L	61	2018/10/20	R
18	2014/10/10	L	62	2018/10/20	R
19	2014/10/10	R	63	2018/10/20	L
20	2014/10/10	R	64	2018/10/20	R
21	2014/10/10	R	65	2018/10/20	L
22	2014/10/10	R	66	2018/10/20	L
23	2014/10/10	R	67	2018/10/20	R
24	2014/10/10	R	68	2018/10/20	R
25	2014/10/10	R	69	2019/06/06	L
26	2014/10/10	R	70	2019/06/06	L
27	2014/10/10	R	71	2020/03/22	L
28	2014/10/10	L	72	2020/03/22	R
29	2014/10/10	R	73	2020/03/22	R
30	2014/10/10	L	74	2021/10/07	L
31	2014/10/10	R	75	2021/10/07	R
32	2014/10/10	L	76	2021/10/07	R
33	2014/10/10	L	77	2021/10/07	L
34	2014/10/10	L	78	2021/10/07	R
35	2014/10/10	L	79	2021/10/07	R
36	2014/10/10	L	80	2025/04/19	L
37	2014/10/10	R	81	2025/04/19	L
38	2014/10/10	R	82	2025/04/19	L
39	2014/10/10	R	83	2025/04/19	L
40	2014/10/10	R	84	2025/04/19	R
41	2014/10/10	R	85	2025/04/19	L
42	2014/10/10	L	86	2025/04/19	R
43	2014/10/10	L	87	2025/04/19	R
44	2014/10/10	R	88	2025/04/19	L

**TABLE A2:** iNaturalist observation URLs for *Trimeresurus stejnegeri* photographs used in this study, with ambush‐posture direction classified as left‐curved (L) or right‐curved (R).

No.	iNaturalist link	Laterality
**Tianmu Hiking Trail (TM) (*n* = 21)**		
1	https://www.inaturalist.org/observations/317783925	R
2	https://www.inaturalist.org/observations/314701501	R
3	https://www.inaturalist.org/observations/314117597	L
4	https://www.inaturalist.org/observations/314078635	L
5	https://www.inaturalist.org/observations/308476392	L
6	https://www.inaturalist.org/observations/278028511	R
7	https://www.inaturalist.org/observations/253973740	L
8	https://www.inaturalist.org/observations/253973634	R
9	https://www.inaturalist.org/observations/250647665	R
10	https://www.inaturalist.org/observations/245987434	L
11	https://www.inaturalist.org/observations/241642209	R
12	https://www.inaturalist.org/observations/198563509	R
13	https://www.inaturalist.org/observations/195168637	R
14	https://www.inaturalist.org/observations/152530491 #1	L
15	https://www.inaturalist.org/observations/152530491 #2	R
16	https://www.inaturalist.org/observations/140851383	L
17	https://www.inaturalist.org/observations/137764304	L
18	https://www.inaturalist.org/observations/137408268	R
19	https://www.inaturalist.org/observations/98776452	R
20	https://www.inaturalist.org/observations/17283072	R
21	https://www.inaturalist.org/observations/13799784	R
**Yangmingshan National Park (YM) (*n* = 38)**		
1	https://www.inaturalist.org/observations/286228370	L
2	https://www.inaturalist.org/observations/254233889	R
3	https://www.inaturalist.org/observations/241844741	R
4	https://www.inaturalist.org/observations/240346601	R
5	https://www.inaturalist.org/observations/233708671	R
6	https://www.inaturalist.org/observations/228319398	L
7	https://www.inaturalist.org/observations/223850195	L
8	https://www.inaturalist.org/observations/215029987	L
9	https://www.inaturalist.org/observations/212200146	R
10	https://www.inaturalist.org/observations/179650092	R
11	https://www.inaturalist.org/observations/173993085	R
12	https://www.inaturalist.org/observations/169206903	L
13	https://www.inaturalist.org/observations/164947247	L
14	https://www.inaturalist.org/observations/126174144	R
15	https://www.inaturalist.org/observations/120946566	R
16	https://www.inaturalist.org/observations/115521977	R
17	https://www.inaturalist.org/observations/57134384	R
18	https://www.inaturalist.org/observations/14127771	L
19	https://www.inaturalist.org/observations/76456300	L
20	https://www.inaturalist.org/observations/312699483	L
21	https://www.inaturalist.org/observations/303103637	R
22	https://www.inaturalist.org/observations/292133909	R
23	https://www.inaturalist.org/observations/291666118	R
24	https://www.inaturalist.org/observations/288957392	R
25	https://www.inaturalist.org/observations/287550951	R
26	https://www.inaturalist.org/observations/248555067	R
27	https://www.inaturalist.org/observations/233472050	L
28	https://www.inaturalist.org/observations/226807705	R
29	https://www.inaturalist.org/observations/226383360	R
30	https://www.inaturalist.org/observations/223850169	R
31	https://www.inaturalist.org/observations/214965484	R
32	https://www.inaturalist.org/observations/204527368	R
33	https://www.inaturalist.org/observations/192877540	L
34	https://www.inaturalist.org/observations/132796884	L
35	https://www.inaturalist.org/observations/63471305	R
36	https://www.inaturalist.org/observations/45461693	R
37	https://www.inaturalist.org/observations/32320429	R
38	https://www.inaturalist.org/observations/99929520	R
**Northern Cross‐Island Highway (HW) (*n* = 103)**		
1	https://www.inaturalist.org/observations/320378425	R
2	https://www.inaturalist.org/observations/320378227	L
3	https://www.inaturalist.org/observations/320377985	R
4	https://www.inaturalist.org/observations/320377564	L
5	https://www.inaturalist.org/observations/318859530	R
6	https://www.inaturalist.org/observations/308728031	L
7	https://www.inaturalist.org/observations/308727971	L
8	https://www.inaturalist.org/observations/308727931	R
9	https://www.inaturalist.org/observations/304717151	L
10	https://www.inaturalist.org/observations/301865825	L
11	https://www.inaturalist.org/observations/294601987	L
12	https://www.inaturalist.org/observations/294601969	R
13	https://www.inaturalist.org/observations/285840812	L
14	https://www.inaturalist.org/observations/281806161	R
15	https://www.inaturalist.org/observations/278811527	R
16	https://www.inaturalist.org/observations/277670895	R
17	https://www.inaturalist.org/observations/261341053	R
18	https://www.inaturalist.org/observations/254100680	R
19	https://www.inaturalist.org/observations/254100658	R
20	https://www.inaturalist.org/observations/246433418	L
21	https://www.inaturalist.org/observations/246433413	R
22	https://www.inaturalist.org/observations/246433411	L
23	https://www.inaturalist.org/observations/246433407	L
24	https://www.inaturalist.org/observations/246433403	L
25	https://www.inaturalist.org/observations/246433400	R
26	https://www.inaturalist.org/observations/246433397	L
27	https://www.inaturalist.org/observations/246433391	R
28	https://www.inaturalist.org/observations/246433389	R
29	https://www.inaturalist.org/observations/244123082	R
30	https://www.inaturalist.org/observations/243885330	L
31	https://www.inaturalist.org/observations/216718799	R
32	https://www.inaturalist.org/observations/202130945	L
33	https://www.inaturalist.org/observations/201897600	R
34	https://www.inaturalist.org/observations/190941998	R
35	https://www.inaturalist.org/observations/189420660	L
36	https://www.inaturalist.org/observations/189202790	L
37	https://www.inaturalist.org/observations/188791068	L
38	https://www.inaturalist.org/observations/188790994	R
39	https://www.inaturalist.org/observations/188790400	L
40	https://www.inaturalist.org/observations/178370666	R
41	https://www.inaturalist.org/observations/178370557	L
42	https://www.inaturalist.org/observations/178370296	R
43	https://www.inaturalist.org/observations/152530640	R
44	https://www.inaturalist.org/observations/129118299	L
45	https://www.inaturalist.org/observations/129118297	L
46	https://www.inaturalist.org/observations/125471560	R
47	https://www.inaturalist.org/observations/122177964	R
48	https://www.inaturalist.org/observations/110722296	L
49	https://www.inaturalist.org/observations/97009309	L
50	https://www.inaturalist.org/observations/94385099	L
51	https://www.inaturalist.org/observations/58950110	L
52	https://www.inaturalist.org/observations/48229562	L
53	https://www.inaturalist.org/observations/45858115	R
54	https://www.inaturalist.org/observations/35525224	L
55	https://www.inaturalist.org/observations/35525221	R
56	https://www.inaturalist.org/observations/33295513	R
57	https://www.inaturalist.org/observations/32879742	L
58	https://www.inaturalist.org/observations/32879741	R
59	https://www.inaturalist.org/observations/26741549	L
60	https://www.inaturalist.org/observations/17730970	L
61	https://www.inaturalist.org/observations/17730968	R
62	https://www.inaturalist.org/observations/17730966	R
63	https://www.inaturalist.org/observations/17730954	R
64	https://www.inaturalist.org/observations/17730948	L
65	https://www.inaturalist.org/observations/17730945	L
66	https://www.inaturalist.org/observations/17730938	L
67	https://www.inaturalist.org/observations/17730930	R
68	https://www.inaturalist.org/observations/17730928	L
69	https://www.inaturalist.org/observations/17730925	L
70	https://www.inaturalist.org/observations/17730893	L
71	https://www.inaturalist.org/observations/17730885	L
72	https://www.inaturalist.org/observations/17730882	R
73	https://www.inaturalist.org/observations/17730876	L
74	https://www.inaturalist.org/observations/17730871	R
75	https://www.inaturalist.org/observations/17730870	R
76	https://www.inaturalist.org/observations/17730867	L
77	https://www.inaturalist.org/observations/17730862	L
78	https://www.inaturalist.org/observations/17730858	L
79	https://www.inaturalist.org/observations/17730855	L
80	https://www.inaturalist.org/observations/17730844	L
81	https://www.inaturalist.org/observations/17730836	R
82	https://www.inaturalist.org/observations/17730826	L
83	https://www.inaturalist.org/observations/17730823	L
84	https://www.inaturalist.org/observations/17730819	L
85	https://www.inaturalist.org/observations/17730814	L
86	https://www.inaturalist.org/observations/17730803	L
87	https://www.inaturalist.org/observations/17730800	R
88	https://www.inaturalist.org/observations/17730795	L
89	https://www.inaturalist.org/observations/17730793	R
90	https://www.inaturalist.org/observations/17730788	L
91	https://www.inaturalist.org/observations/17730787	L
92	https://www.inaturalist.org/observations/17730786	R
93	https://www.inaturalist.org/observations/17730785	R
94	https://www.inaturalist.org/observations/17730784	R
95	https://www.inaturalist.org/observations/17730783	L
96	https://www.inaturalist.org/observations/17730780	R
97	https://www.inaturalist.org/observations/17730777	L
98	https://www.inaturalist.org/observations/17730774	R
99	https://www.inaturalist.org/observations/17730772	L
100	https://www.inaturalist.org/observations/17730771	L
101	https://www.inaturalist.org/observations/17730767	L
102	https://www.inaturalist.org/observations/13975022	L
103	https://www.inaturalist.org/observations/13931571	R

**TABLE A3:** Generalized linear model (GLM) for ambush‐posture direction (right‐curved vs. left‐curved) at Yangmingshan (*n* = 51).

Variables	Estimate	SE	*z*	*p*	95% CI
Intercept	−159.6423	259.431	−0.615	0.538	−668.118, 348.833
Age (juvenile vs. adult)	0.5505	1.032	0.533	0.594	−1.473, 2.574
Perching height (high vs. low)	0.3671	0.795	0.462	0.644	−1.191, 1.926
Season (autumn vs. summer)	0.5127	0.931	0.551	0.582	−1.312, 2.338
Season (spring vs. summer)	−0.0170	0.930	−0.018	0.985	−1.840, 1.807
Season (winter vs. summer)	16.0185	1455.398	0.011	0.991	−2836.509, 2868.546
Data source (field survey vs. citizen science)	0.5671	0.685	0.828	0.408	−0.776, 1.910
Year	0.0783	0.128	0.610	0.542	−0.173, 0.330

## References

[ece373627-bib-0001] Amano, M. , Y. Kawano , T. Kubo , T. Kuwahara , and H. Kobayashi . 2021. “Population‐Level Laterality in Foraging Finless Porpoises.” Scientific Reports 11: 21164. 10.1038/s41598-021-00635-6. 34707173 PMC8551196

[ece373627-bib-0002] Anderson, H. M. , D. N. Fisher , B. L. McEwen , J. Yeager , J. N. Pruitt , and J. B. Barnett . 2021. “Episodic Correlations in Behavioural Lateralization Differ Between a Poison Frog and Its Mimic.” Animal Behaviour 174: 207–215. 10.1016/j.anbehav.2021.01.011.

[ece373627-bib-0003] Berlinghieri, F. , P. Panizzon , I. L. Penry‐Williams , and C. Brown . 2021. “Laterality and Fish Welfare ‐ A Review.” Applied Animal Behaviour Science 236: 105239. 10.1016/j.applanim.2021.105239.

[ece373627-bib-0004] Bonati, B. , D. Csermely , and R. Romani . 2008. “Lateralization in the Predatory Behaviour of the Common Wall Lizard ( *Podarcis muralis* ).” Behavioural Processes 79: 171–174. 10.1016/j.beproc.2008.07.007. 18703120

[ece373627-bib-0005] Brown, C. , C. Gardner , and V. A. Braithwaite . 2004. “Population Variation in Lateralized Eye Use in the Poeciliid *Brachyraphis episcopi* .” Proceedings of the Royal Society of London ‐ Series B: Biological Sciences 271: S455–S457. 10.1098/rsbl.2004.0222. PMC1810090 15801602

[ece373627-bib-0006] Cashmore, L. , N. Uomini , and A. Chapelain . 2008. “The Evolution of Handedness in Humans and Great Apes: A Review and Current Issues.” Journal of Anthropological Sciences 86: 7–35. https://www.isita‐org.com/jass/Contents/2008%20vol86/03_Cashmore.pdf. 19934467

[ece373627-bib-0007] Chang, K. X. , B. H. Huang , M. X. Luo , et al. 2021. “Niche Partitioning Among Three Snail‐Eating Snakes Revealed by Dentition Asymmetry and Prey Specialisation.” Journal of Animal Ecology 90: 967–977. 10.1111/1365-2656.13426. 33481265

[ece373627-bib-0008] Chen, Y. H. 2023. Survey and Monitoring Project on Invasive Amphibian Species in Yangmingshan National Park. Yangmingshan National Park.

[ece373627-bib-0009] Creer, S. , W. H. Chou , A. Malhotra , and R. S. Thorpe . 2002. “Offshore Insular Variation in the Diet of the Taiwanese Bamboo Viper *Trimeresurus stejnegeri* (Schmidt).” Zoological Science 19: 907–913. 10.2108/zsj.19.907. 12193807

[ece373627-bib-0010] Csermely, D. , B. Bonati , and R. Romani . 2010. “Lateralisation in a Detour Test in the Common Wall Lizard ( *Podarcis muralis* ).” Laterality 15: 535–547. 10.1080/13576500903051619. 19739021

[ece373627-bib-0011] Dill, L. M. 1977. “‘Handedness’ in the Pacific Tree Frog ( *Hyla regilla* ).” Canadian Journal of Zoology 55: 1926–1929. 10.1139/z77-248.

[ece373627-bib-0012] Friedlaender, A. S. , J. E. Herbert‐Read , E. L. Hazen , et al. 2017. “Context‐Dependent Lateralized Feeding Strategies in Blue Whales.” Current Biology 27: R1206–R1208. 10.1016/j.cub.2017.10.023. 29161554

[ece373627-bib-0013] Heatwole, H. , P. King , and S. G. Levine . 2007. “Laterality in Coiling Behaviour of Snakes: Another Interpretation.” Laterality 12: 536–542. 10.1080/13576500701602944. 17852698

[ece373627-bib-0014] Herrel, A. , K. Huyghe , P. Oković , D. Lisičić , and Z. Tadić . 2011. “Fast and Furious: Effects of Body Size on Strike Performance in an Arboreal Viper *Trimeresurus* (*Cryptelytrops*) *albolabris* .” Journal of Experimental Zoology Part A 315: 22–29. 10.1002/jez.645. 20853417

[ece373627-bib-0015] Honan, C. , and C. M. Murray . 2023. “The Effect of Androgen Exposure on Cerebral Lateralization in the American Alligator ( *Alligator mississippiensis* ).” General and Comparative Endocrinology 336: 114248. 10.1016/j.ygcen.2023.114248. 36848983 PMC10071487

[ece373627-bib-0016] Hopkins, W. D. 2006. “Comparative and Familial Analysis of Handedness in Great Apes.” Psychological Bulletin 132: 538–559. 16822166 10.1037/0033-2909.132.4.538 PMC2063575

[ece373627-bib-0017] Hoso, M. 2017. “Asymmetry of Mandibular Dentition Is Associated With Dietary Specialization in Snail‐Eating Snakes.” PeerJ 5: e3011. 10.7717/peerj.3011. 28265502 PMC5337109

[ece373627-bib-0018] Hoso, M. , T. Asami , and M. Hori . 2007. “Right‐Handed Snakes: Convergent Evolution of Asymmetry for Functional Specialization.” Biology Letters 3: 169–173. 10.1098/rsbl.2006.0600. 17307721 PMC2375934

[ece373627-bib-0019] Huang, M. C. W. , C. P. Liao , C. C. Chou , J. W. Lin , and W. S. Huang . 2022. “Size of Snake Eyes Correlates With Habitat Types and Diel Activity Patterns.” Frontiers in Ecology and Evolution 9: 821965. 10.3389/fevo.2021.821965.

[ece373627-bib-0020] Jayne, B. C. 2020. “What Defines Different Modes of Snake Locomotion?” Integrative and Comparative Biology 60: 156–170. 10.1093/icb/icaa017. 32271916 PMC7391877

[ece373627-bib-0021] Karenina, K. , and A. Giljov . 2022. “Lateralization in Feeding Is Food Type Specific and Impacts Feeding Success in Wild Birds.” Ecology and Evolution 12: e8598. 10.1002/ece3.8598. 35154659 PMC8820115

[ece373627-bib-0022] Keuroghlian‐Eaton, S. , S. P. dos Santos , M. Marrie Pinheiro Müller , D. G. Cavalheri , B. E. Bolochio , and D. J. Santana . 2024. “Escape Behavior Lateralization of Pointed‐Belly Frog ( *Leptodactylus podicipinus* ) (Anura, Leptodactylidae) in the Southern Pantanal.” Biotropica 56: e13326. 10.1111/btp.13326.

[ece373627-bib-0023] Lippolis, G. , A. Bisazza , L. J. Rogers , and G. Vallortigara . 2002. “Lateralisation of Predator Avoidance Responses in Three Species of Toads.” Laterality 7: 163–183. 10.1080/13576500143000221. 15513195

[ece373627-bib-0024] Morita, K. , Y. Guo , and T. Toi . 2023. “Age‐Related Asymmetry in Left‐Right Ears of Sound Lateralization With Respect to Four Different Rise Times.” Frontiers in Neuroscience 17: 1249119. 10.3389/fnins.2023.1249119. 37732306 PMC10508348

[ece373627-bib-0026] Poo, W. K. , J. F. Chen , S. C. Kuo , et al. 2003. “Studies of Fauna and Flora Around Yangmingshan—Tianmu Ancient Trail.” Huagang Journal of Science 20: 27–70. https://www.airitilibrary.com/Article/Detail?DocID=a0000027‐200305‐x‐20‐27‐70‐a.

[ece373627-bib-0027] Raymond, M. , and D. Pontier . 2004. “Is There Geographical Variation in Human Handedness?” Laterality 9: 35–51. 10.1080/13576500244000274. 15382729

[ece373627-bib-0028] Robins, A. , P. Chen , L. D. Beazley , and S. A. Dunlop . 2005. “Lateralized Predatory Responses in the Ornate Dragon Lizard ( *Ctenophorus ornatus* ).” Neuroreport 16: 849–852. 15891583 10.1097/00001756-200505310-00014

[ece373627-bib-0029] Robins, A. , and L. J. Rogers . 2004. “Lateralized Prey‐Catching Responses in the Cane Toad, *Bufo marinus* : Analysis of Complex Visual Stimuli.” Animal Behaviour 68: 767–775. 10.1016/j.anbehav.2003.12.014.

[ece373627-bib-0030] Robins, A. , and L. J. Rogers . 2006. “Lateralized Visual and Motor Responses in the Green Tree Frog, *Litoria caerulea* .” Animal Behaviour 72: 843–852. 10.1016/j.anbehav.2006.01.022.

[ece373627-bib-0031] Rogers, L. J. 2021. “Brain Lateralization and Cognitive Capacity.” Animals 11: 1996. 10.3390/ani11071996. 34359124 PMC8300231

[ece373627-bib-0032] Roth, E. D. 2003. “‘Handedness’ in Snakes? Lateralization of Coiling Behaviour in a Cottonmouth, *Agkistrodon piscivorus leucostoma* , Population.” Animal Behaviour 66: 337–341. 10.1006/anbe.2003.2228.

[ece373627-bib-0033] Shine, R. , M. M. Olsson , M. P. LeMaster , I. T. Moore , and R. T. Mason . 2000. “Are Snakes Right‐Handed? Asymmetry in Hemipenis Size and Usage in Gartersnakes ( *Thamnophis sirtalis* ).” Behavioral Ecology 11: 411–415. 10.1093/beheco/11.4.411.

[ece373627-bib-0034] Shine, R. , and L. X. Sun . 2003. “Attack Strategy of an Ambush Predator: Which Attributes of the Prey Trigger a Pit‐Viper's Strike?” Functional Ecology 17: 340–348. 10.1046/j.1365-2435.2003.00738.x.

[ece373627-bib-0035] Singh, M. , and M. P. Bryden . 1994. “The Factor Structure of Handedness in India.” International Journal of Neuroscience 74: 33–43. 10.3109/00207459408987227. 7928113

[ece373627-bib-0036] Tsai, T. S. , and M. C. Tu . 1998. “Sexual Dimorphism of Chinese Green Tree Viper, *Trimeresurus stejnegeri stejnegeri* .” Biological Bulletin of National Taiwan Normal University 33: 13–22.

[ece373627-bib-0037] Tsai, T. S. , and M. C. Tu . 2000. “Reproductive Cycle of Male Chinese Green Tree Vipers, *Trimeresurus s. stejnegeri*, in Northern Taiwan.” Journal of Herpetology 34: 424–430. 10.2307/1565366.

[ece373627-bib-0038] Uziel, D. , M. Lopes‐Conceição , D. Simpson , and R. Lent . 1998. “Ontogenesis of Lateralized Rotational Behavior in Hamsters: A Time Series Study.” Behavioural Brain Research 92: 47–53. 10.1016/S0166-4328(97)00122-8. 9588684

[ece373627-bib-0039] van Soldt, B. J. , B. D. Metscher , R. E. Poelmann , et al. 2015. “Heterochrony and Early Left‐Right Asymmetry in the Development of the Cardiorespiratory System of Snakes.” PLoS One 10: e116416. 10.1371/journal.pone.0116416. 25555231 PMC4282204

[ece373627-bib-0040] Xu, Y. , J. Deng , T. Zhang , et al. 2025. “A ‘Little Dragon’ From Kunming City: A New Green Pit Viper From Yunnan Province, China (Squamata, Viperidae, *Trimeresurus*).” Zoosystematics and Evolution 101: 2267–2293. 10.3897/zse.101.175879.

[ece373627-bib-0041] Yang, C. K. , and A. Mori . 2021. “The Green Bamboo Pit Viper, *Trimeresurus stejnegeri* , Discriminates Chemical Stimuli Among Anuran Species.” Current Herpetology 40: 159–168. 10.5358/hsj.40.159.

[ece373627-bib-0042] Yang, C. K. , Y. J. Yang , and A. Mori . 2024. “Ambush Site Selection by a Green Bamboo Pit Viper: Relation to Prey Abundance and Comparison Between Juveniles and Adults.” Zoological Studies 63: e55. 10.6620/zs.2024.63-55. 40933663 PMC12417141

[ece373627-bib-0043] You, C. W. , and J. C. Wang . 2007. “A Preliminary Survey of Amphibian and Reptile Resources Along the Northern Cross‐Island Highway.” Nature Conservation Quarterly 59: 38–47. 10.29738/ncq.200709.0007.

